# Economical implications and the impact of gonadotropin-releasing hormone administration at the time of artificial insemination in cows raised in the extensive system in North Romania

**DOI:** 10.3389/fvets.2023.1167387

**Published:** 2023-04-27

**Authors:** Daniel Berean, Liviu Marian Bogdan, Petrică Opris, Raluca Cimpean

**Affiliations:** ^1^Department of Reproduction, Faculty of Veterinary Medicine, University of Agricultural Sciences and Veterinary Medicine from Cluj-Napoca, Cluj-Napoca, Romania; ^2^CSV Barsana, Maramureş County, Romania

**Keywords:** artificial insemination, gonadotropin-releasing hormone, extensive system, North Romania, fertility rate, economical implications

## Abstract

Artificial insemination (AI) is the first and the most used biotechnologies in reproduction all over the world. Many studies reported the beneficial role of gonadotropin-releasing hormone (GnRH) administrated some hours before the AI or at the artificial insemination time. This study aimed to assess the effect of GnRH analogs given at the time of insemination on the first, second, and third AIs and to assess the economical implications of GnRH administration. We hypothesized that administration of GnRH at the time of insemination would increase ovulation and pregnancy rate. The study was conducted on small farms in northwestern Romania and included animals of the Romanian Brown and Romanian Spotted breeds. Animals in estrous at the first, second, and third inseminations were randomly divided into groups that received GnRH at insemination and groups that did not. A comparison between the groups was performed and the cost of GnRH administration to obtain one gestation was calculated. The GnRh administration increased the pregnancy rate at the first and the second inseminations by 12 and 18%, respectively. For one pregnancy, the cost of GnRH administration was approximately 49 euros for the first insemination group and around 33 euros for the second insemination group. No improvement of the pregnancy rate was observed after the GnRH administration for the cows at the third insemination, so, for this group, no economic statistics were performed.

## 1. Introduction

Artificial insemination is the first and the most used biotechnologies in reproduction all over the world. One of the main objectives for both dairy and beef farm cows is to have high reproductive performances (1). Therefore, the viability of cow production systems is limited by reproductive inefficiency, which causes financial losses for cattle producers (2). The fertility rate is the primary indication of reproductive performance, and the establishment of pregnancy following insemination is the primary definition of fertility in most dairy systems (3). Another important reproductive indicator for the cattle system is the pregnancy rate, which we may define as the percentage of eligible cows that become pregnant within a 21-day period (4). A similar characteristic to the pregnancy rate is the first-service conception rate. The conception rate is determined by the number of cows inseminated over a 21-day period divided by the percentage of cows that are pregnant (5). The economic health of the farm is directly impacted by the cows' first-service conception rate. Although the percentage success of the first service ranged from 26.7 to 50.7% in prior research (6, 7), conception during the first service after calving is crucial for cows to operate at their best reproductively. The expense of semen and the work required for heat checking and breeding for numerous AI are modest for this group (8). Factors such as body condition score, peripartum disorders (dystocia, metritis, and retained placenta), cow parities, higher milk yield (>39 kg/day), clinical ketosis, and twin births were associated with low conception at the first service (9–11).

Numerous studies have documented the beneficial effects of administering gonadotropin-releasing hormone (GnRH) at or just before the time of AI (12–16). It was postulated that the administration of a single dose of GnRH synchronizes the preovulatory LH surge and ovulation with the timing of AI. The administration of a GnRH analog at estrus increases peak LH surge and is consequently related to higher progesterone levels after ovulation (12, 13). Moreover, numerous studies (17–20) showed that the luteal phase of the estrus cycle benefited from GnRH hormonal stimulation.

The majority of cattle in Romania are raised in extensive breeding systems with tie-stalls; in small farms with a number of cows <30, and in medium-sized farms with a number of cows <150 (21). During the colder months of the year (5–6 months per year), cows are kept permanently tied in stalls, under shelters, while during the warmer months of the year (6–7 months per year), they are allowed to graze freely all day (21). The average number of cattle per 100 ha in the northwest area was 20.52 in 2018 (22), with the dominating breeds being the Romanian Brown and Romanian Spotted.

This study aimed to assess the effect of GnRH analogs given at the time of AI on the first, second, and third AIs and the economical implication of the GnRH administration. The study involved both Romanian brown and Romanian spotted breeds of cattle, and it was conducted on small farms in northwest Romania.

## 2. Materials and methods

**Figure 1:**
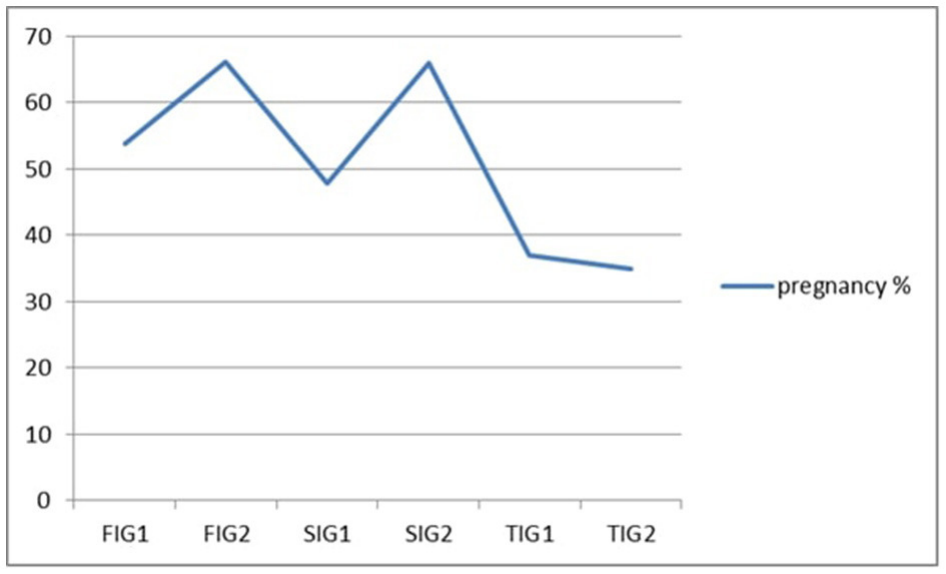
Pregnancy rate for each group.

**Figure 2:**
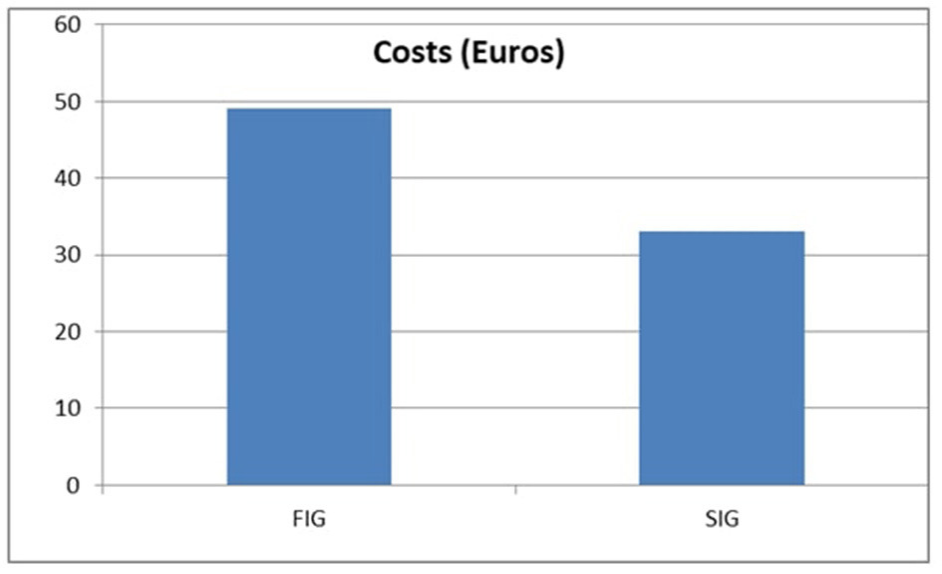
Cost with GnRH administration to obtain 1 pregnancy.

The study was carried out on small farms in northwestern Romania (*n* = 58), farms with several animals that range between 3 and 124. The data were collected from three AI operators that performed 1,322 inseminations in the interval between January 2022 and October 2022. The cows that were included in the study were females with condition body scores between 2.5 and 3.5 out of 5 (23), no postpartum pathologies, and at least 45 days after parturition. Before the insemination, each animal underwent a clinical examination, and a clinically healthy diagnosis was established, the animals with intermittent estrus to bull-like behavior, such as mounting, pawing the ground, and bellowing, as well as those with purulent or mucopurulent discharge, were not included in this research. The number of parturitions was not a factor in the selection of the cows, all groups had cows with parturitions ranging from one to four. All the animals were followed with the aim to determine the percent of gestation at the first, second, and third inseminations and were included in three groups: first insemination group (FIG, *n* = 420), second insemination group (SIG, *n* = 610), and third insemination group (TIG, *n* = 292). All three groups were equally and randomly divided into another two groups. FIG was divided into FIG 1 (*n* = 210) and FIG 2 (*n* = 210); for the animals included in FIG 1, no treatment was performed on the AI day, and for the animals included in FIG 2, 100 μg D-Phe 6-gonadorelin (2 ml i.m, Gonavet, Veyx-Pharma GmbH) were administrated at the AI time. SIG was divided into Sig 1 (*n* = 305) and Sig 2 (*n* = 305). No treatment was performed for Sig 1 group at the AI time, and 100 μg D-Phe 6-gonadorelin (2 ml i.m, Gonavet, Veyx-Pharma GmbH) was administrated for Sig 2 group at the AI time. TIG was divided into TIG 1 (*n* = 146) and TIG 2 (*n* = 146); for TIG 1, no treatment at the AI time, and 100 μg D-Phe 6-gonadorelin (2 ml i.m, Gonavet, Veyx-Pharma GmbH) for TIG 2 group at the AI time (Table 1).

**Table 1:** Groups and results.

**Groups**	**First insemination group (*****n =*** **420)**		**Second insemination group (*****n =*** **610)**		**Third insemination group (** ***n =*** **292)**	
Groups	FIG1 (*n =* 210)	FIG 2 (*n =* 210)	SIG 1 (*n =* 305)	SIG 2 (*n =* 305)	TIG1 (*n =* 146)	TIG2 (*n =* 146)
Hormonal stimulation at AI	-	100 μg D-Phe 6-gonadorelin	-	100 μg D-Phe 6-gonadorelin	-	100 μg D-Phe 6-gonadorelin
Pregnancy rate (Number/percent)	113/54%	139/66%	146/48%	201/66%	54/37%	51/35%
CG	49 Euros		33 Euros		-	

The insemination was performed after clinical visual observation of some of the following estrus signs: mounting or attempting to mount other cattle, standing to be mounted by other cattle, smelling other females, following up other females, bellowing, depressed appetite, nervous and excitable behavior, mud on hindquarters and sides of cattle, roughed up tail hair, vulva swelling and reddening, clear vaginal mucous discharge, and mucous smeared on the rump. The insemination was performed approximately 8–12 h after the estrus detection.

For all groups (*n* = 6) was estabilished the percent of gestation, and the pregnancy was diagnosed by ultrasonography 35–45 days after the insemination. Females diagnosed empty from FIG 1 and FIG 2 groups were included in the following groups, SIG groups and TIG groups, respectively. Empty animals in FIG 2 and SIG 2 were not included in other groups.

We took an injection cost of 6 euros to calculate the financial effects of the GnRH administration; typically, the cost of one administration of GnRH in our country ranges from 6 to 12 euros. The following calculation was used to determine the GnRH administration's benefit:

CG = IG2 × 6:Pd

Where,

CG (Euros) = cost for 1 gestation

6 (Euros) = price for GnRH administration

IG2 (number) = number of inseminated animals for the second group (FIG2, SIG2, TIG2)

Pd (number) = pregnancy differences between Group 2 and 1 (FIG2-FIG1, SIG2-SIG1)

All data were entered into Microsoft Excel and statistically analyzed to demonstrate the favorable impact of GnRH at the AI time on small farms in the northwest of Romania. A comparison between the groups was done. To compare the groups and find the significant variation, the *t*-test was used.

## 3. Results

The overall pregnancy rate in this research was 53% (704/1320), 47% (313/661) for the groups in which ovarian stimulation with GnRH was not conducted, and 59% (391/661) for the groups where it was performed.

### 3.1. First insemination groups

In the case of the cows at the first insemination, the GnRH hormonal stimulation increased the pregnancy rate. From 210 cows who were artificially inseminated without the use of GnRH (group FIG1), 113 animals (54%) had gestation confirmed. Of the 210 animals who were inseminated for the first time using GnRH injection (group FIG2), 139 (66%) had gestation confirmed (Table 1, Figure 1). After comparing these two groups, a significant difference (*t*-test value of 0.0045 at *p* = 0.5) was found.

The economic statistics showed that for improving the pregnancy with one gestation, it is necessary to spend approximately 49 Euros.

### 3.2. Second insemination groups

The administration of GnRH at the AI time in the case of second insemination was likewise linked to a rise in the pregnancy rate. From 305 inseminations performed for the SIG1 group, a total number of 146 pregnancies (48%) was obtained. In the case of the SIG2 group, in which 201 pregnancies were obtained from 305 inseminations, the rate of pregnancy was 66% (Table 1, Figure 1). A significant difference was also obtained after the *t*-test determination (0.00011 at *p* < 0.5). The cost to improve the pregnancy rate with one gestation for the SIG2 group was somewhere around 33 Euros.

### 3.3. Third insemination groups

In our investigation, the third insemination did not have the same effects as the first two groups when GnRH was administered. The pregnancy rate for the TIG1 group was 37%, with 54 of the 146 inseminated cows having gestations that were confirmed. Pregnancy was confirmed in 51 cases in the TIG2 group, which had a slightly lower pregnancy rate (35%) than the TIG 1 group (Table 1). No significant differences were obtained (*t*-test value of 0.35 at *p* < 0.5) after the comparison of these two groups. This group did not have economic statistics run because there was no improvement in the pregnancy rate.

## 4. Discussion

To achieve the goal of annual calving in cattle, the open days may only be split by a maximum of 90 days (24). Numerous tactics were employed to boost the rate of conception during the initial insemination in order to accomplish this goal (25–28). In earlier research, GnRH and human chorionic gonadotropin (hCG) were utilized to improve ovulation and conception rates (29, 30).

According to Kim and Jeong (31), cows that failed to conceive required more money to be spent on reproductive treatment ($55.40) and other management ($567.00) than cows that conceived after the first insemination. These findings suggest that GnRH treatment at the first AI is a wise choice for the farmers when balanced against the cost of GnRH administration in our study.

Similar to the results of our investigation, Kaim et al. (14) observed an increase in conception rate following injection of GnRH, from 36.0 to 61.5% in cows with lower body condition and from 42.2 to 63.2% in primiparous cows. The difference in conception rates between the treated group and the non-treated group in the current study was less evident. The explanation can be the fact that in our study the animals were selected randomly, and we did not choose groups with small reproduction indices (cows with lower body condition/primiparous cows).

The second insemination may still provide a chance to meet the aim of one calf per year if there is appropriate reproductive management (heat detection and insemination) on the farm. In our study, the pregnancy rate for the second insemination group was similar to that of the first insemination group (66%), but it was lower for the unstimulated group (SIG1) (48 vs. 54%). When GnRH was administered during AI, the pregnancy rate for the SIG2 group vs. the SIG 1 group increased considerably (*t*-test value 0.000011). The price with the GnRH administration was less than for the first group, at roughly 33 Euros (Figure 2).

The pregnancy improvement and the cost of the GnRH administration demonstrate that the second insemination cows gain more from the GnRH administration than the first insemination cows do. Hubner et al. (32) observed no benefits of GnRH treatment at the A.I. time for cows diagnosed in estrus by traditional estrus detection or an automated activity-monitoring system, they reported pregnancy rates between 35 and 43% for treated cows and no treated cows. The benefit of GnRH administration can be attributed to two factors: cows detected in estrus who fail to ovulate, 6.5–44% (33, 34), or cows who have delayed ovulation (>35 h after estrus = 10%) (35). Since cows that do not ovulate are unable to become pregnant, increasing the ovulation rate would have an impact on the conception rate (32). As in our study, Burnett et al. (36) showed that GnRH at the time of AI increased the pregnancy rate for cows found in estrus.

Hubner et al. (32) reported a lower pregnancy rate for cows receiving the first service compared with herd mates receiving ≥2 services. In our study, the lower pregnancy rate was found in the third group (35 and 37%), in the case of the cows at the third insemination. A meta-analysis about the effects of GnRH on conception at the time of insemination published by Morgan and Lean (35) explained that the benefits of GnRH administration at AI were only present for ≥2 services. Valenza et al. (37) were unable to replicate the benefits of GnRH for ≥2 services. We demonstrated, in this study, the benefit of GnRH administration for the first two services, but, in the case of the third service, the treatment did not have the same effect; no differences were observed between the hormonal-stimulated and the non-hormonal-stimulated group (37 vs. 35%).

Repeat breeding (RB), defined as cows' failure to conceive from three or more regularly spaced services in the absence of detectable abnormalities, is a costly problem for cows' farmers (38). Repeat breeding (RB) is a significant issue in cattle breeding that causes significant economic loss since it results in more inseminations, longer calving intervals, and higher culling rates (39). The GnRH administration carried out in our study showed that GnRH is not a good choice in the treatment of RB, as the effectiveness of GnRH administration at the AI time is lost after the second service.

## 5. Conclusion

The pregnancy rate in the group of cows at the first and second services (12 and 18%) increased as a result of the administration of GnRH at the time of AI. The cost with the GnRH administration to obtain one pregnancy was 49 Euros at the first insemination at 33 Euros at the second insemination. In the case of cows at the third service, no advantage was seen following the GnRH administration. In addition, a thorough examination of the reproductive system is required during the third insemination to determine the cause of infertility.

## Data availability statement

The raw data supporting the conclusions of this article will be made available by the authors, without undue reservation.

## Ethics statement

The animal study was reviewed and approved by the Bioethics Commission of the USAMV CLUJ NAPOCA. Written informed consent was obtained from the owners for the participation of their animals in this study.

## Author contributions

DB and LB: conceptualization, formal analysis, and writing—review and editing. DB and PO: methodology and investigation. RC: statistical analysis, writing, and validation. DB: writing—review and editing. LB: conceptualization, formal analysis, supervision, methodology, and writing—original draft. All authors contributed to the article and approved the submitted version.
